# Seven New Tetrahydroanthraquinones from the Root of *Prismatomeris connata* and Their Cytotoxicity against Lung Tumor Cell Growth

**DOI:** 10.3390/molecules201219856

**Published:** 2015-12-17

**Authors:** Chunxiang Wang, Xiao Ding, Shi-Xiu Feng, Qiunong Guan, Xiao-Ping Zhang, Caigan Du, Ying-Tong Di, Tao Chen

**Affiliations:** 1 Key Laboratory of South Subtropical Plant Diversity, Fairy Lake Botanical Garden, Shenzhen & Chinese Academy of Sciences, Shenzhen 518004, China; cxwang2015@hotmail.com (C.W.); fengshixiu@scbg.ac.cn (S.-X.F.); 2 State Key Laboratory of Phytochemistry and Plant Resources in West China, Kunming Institute of Botany, Chinese Academy of Sciences, Kunming 650204, China; dingxiao@mail.kib.ac.cn; 3 School of Life Sciences, Anhui Normal University, Wuhu 241000, China; pinghengxu@sina.com; 4 Department of Urologic Sciences, University of British Columbia, Vancouver, BC V6H 3Z6, Canada; qiunong@hotmail.com

**Keywords:** natural compound, *Prismatomeris connata*, tetrahydroanthraquinones, prisconnatanones C–I, HPLC-NMR, anti-tumor activity

## Abstract

The root of *Prismatomeris connata* has been used in China for centuries as the medicinal herb “*Huang Gen*” (HG), but its phytochemicals or active ingredients are not well understood. In this study, we performed chemical analysis of the ethyl acetate fraction of a HG ethanol extract. We thus isolated seven new tetrahydroanthraquinones, prisconnatanones C–I (compounds **1**–**7**) from the root of *P. connata* and identified their structures using spectroscopic analyses. Their absolute configurations were established by both modified Mosher’s and Mo_2_OAc_4_ methods, and ORD techniques. Their cytotoxicity was tested in a panel of human lung tumor cells (H1229, HTB179, A549 and H520 cell lines). Prisconnatanone I (**7**) showed the highest activity, with an IC_50_ value ranging from 2.7 µM to 3.9 µM in the suppression of tumor cell growth, and the others with chelated phenolic hydroxyls exhibited relatively lower activity (IC_50_: 8–20 µM). In conclusion, these data suggest that some of the natural tetrahydroanthraquinones in HG are bioactive, and hydroxylation at C-1 significantly increases the cytotoxicity of these compounds against lung tumor cell growth.

## 1. Introduction

The root of *Prismatomeris connate*, termed “*Huang Gen*” (HG) in Chinese herbal medicine, has been used in traditional medicine in China for the treatment of hepatitis, anaemia, leucocythemia, and pneumoconiosis [1,2,3]. Many secondary metabolites, including anthraquinones, anthraquinone glycosides, and iridoids have been identified in HG extracts in earlier studies [4,5,6,7,8,9,10], and in our previous study, two tetrahydroanthraquinones (prisconnatanones A and B) have been successfully isolated, and prisconnatanone A showed a significant cytotoxicity against the A549 lung tumor cell line [9]. This study aimed at further chemical analyses of the phytocomponents in the ethyl acetate (EtOAc) fraction of a HG ethanol extract. In addition to the two known tetrahydroanthraquinone compounds, we identified seven new tetrahydroanthraquinones, prisconnatanones C–I (compounds **1**–**7**). Herein, we report the isolation, structure identification, and biological activities of these new natural compounds.

## 2. Results and Discussion

The 95% EtOH HG extract was successfully fractionated with petroleum ether (PE), ethyl acetate (EtOAc), and *n*-butanol (BuOH). Seven new tetrahydroanthraquinones **1**–**7**, which we have named prisconnatanones C–I, were then purified from the EtOAc soluble fraction by repeated column chromatography on silica gel, Sephadex LH-20, and semipreparative high-performance liquid chromatography (HPLC).

**Figure 1:**
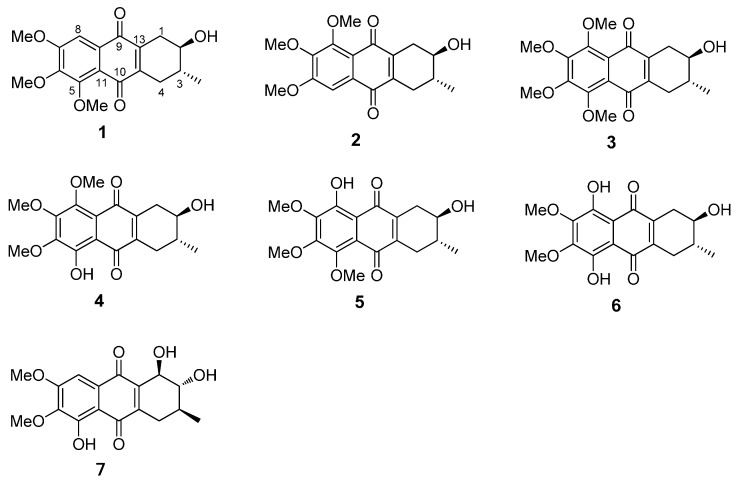
Chemical structure of compounds **1**–**7**.

**Figure 2:**
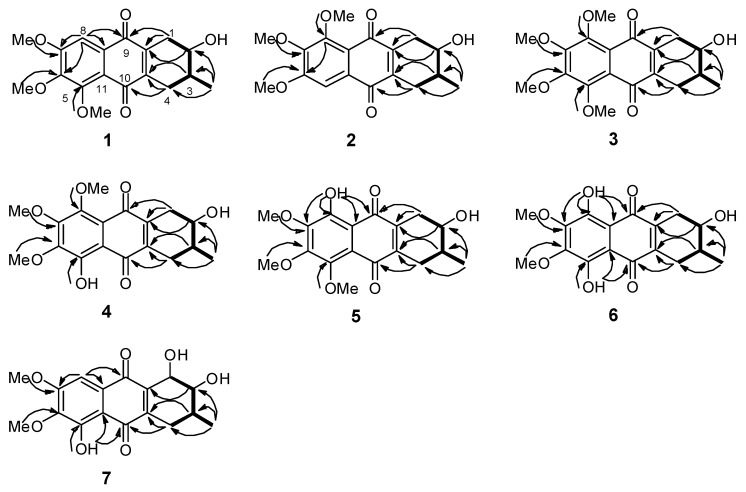
Key ^1^H-^1^H COSY (bold) and HMBC (arrows) correlations of **1**–**7**.

**Figure 3:**
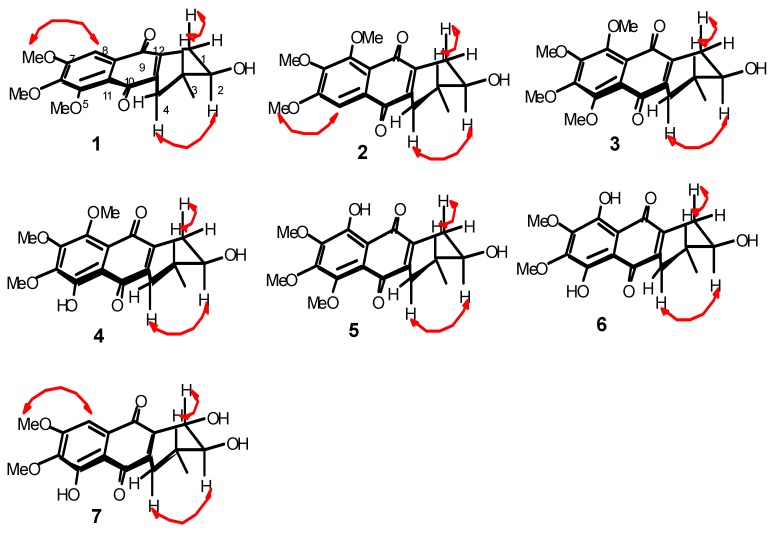
Key ROESY (red arrows) correlations of **1**–**7**.

The chemical properties of these prisconnatanones C–I were determined with both ^13^C-NMR (Table 1) and ^1^H-NMR spectroscopy (Table 2). Their specific chemical structures (Figure 1) were identified according to 1D and 2D NMR spectra (Figure 2 and Figure 3).

**Table 1:** ^13^C-NMR data for prisconnatanones C−I (**1**−**7**) ^a^.

Position	1 ^b^	2 ^b^	3 ^b^	4 ^b^	5 ^b^	6 ^b^	7 ^b^
	δ_C_, mult.	δ_C_, mult.	δ_C_, mult.	δ_C_, mult.	δ_C_, mult.	δ_C_, mult.	δ_C_, mult.
1	30.9, CH2	31.3, CH2	30.9, CH2	31.3, CH2	30.3, CH2	29.8, CH2	73.0, CH
2	71.0, CH	71.1, CH	71.0, CH	70.9, CH	70.8, CH	34.1, CH2	76.7, CH
3	34.2, CH	34.0, CH	34.0, CH	33.8, CH	34.0, CH	71.0, CH2	31.9, CH
4	29.9, CH2	29.4, CH2	29.4, CH2	28.8, CH2	29.9, CH2	31.4, CH2	31.1, CH2
5	154.7, C	106.1, C	152.7, C	154.7, C	149.2, C	159.5, C	155.9, C
6	157.3, C	147.9, C	151.2, C ^c^	146.7, C	153.7, C	147.9, C	141.9, C
7	147.9, C	157.3, C	151.2, C ^c^	153.7, C	146.7, C	148.0, C	158.1, C
8	106.1, C	154.3, C	152.7, C	149.1, C	154.6, C	159.6, C	104.3, CH
9	184.2, C	183.2, C	183.7, C	182.5, C	189.3, C	181.8, C	185.5, C
10	183.3, C	184.2, C	183.7, C	189.4, C	182.6, C	181.9, C	188.7, C
11	129.7, C	119.8, C	121.8, C	110.6, C	118.9, C	107.7, C	111.0, C
12	119.7, C	129.6, C	121.8, C	119.0, C	110.7, C	107.8, C	127.7, C
13	139.8, C	143.6, C	141.8, C	144.7, C	140.3, C	138.3, C	145.1, C
14	145.0, C	141.2, C	143.2, C	141.8, C	146.2, C	137.1, C	142.1, C
CH3 (3)	17.5	17.4	17.4	17.4	17.4	17.6	17.4
OCH3 (5)	61.8		61.8 ^c^		61.6 ^c^		
OCH3 (6)	56.6	61.6	61.9 ^c^	61.9	62.0		61.3
OCH3 (7)	61.6	56.6	61.9 ^c^	61.2	61.6 ^c^	61.9 ^c^	56.7
OCH3 (8)		61.8	61.8 ^c^	61.6		61.9 ^c^	

^a^ The chemical shifts in δ values (ppm) from TMS. ^13^C multiplicities were determined by HSQC assay; ^b^ Readout (δ_C_, mult.) in CDCl_3_ at 150 MHz; ^c^ overlapped.

**Table 2:** ^1^H-NMR Data for prisconnatanones C−I (**1**−**7**) ^a^.

Position	1 ^b^	2 ^b^	3 ^b^	4 ^b^	5 ^b^	6 ^b^	7 ^b^
	δ_H_ (*J* in Hz)	δ_H_ (*J* in Hz)	δ_H_ (*J* in Hz)	δ_H_ (*J* in Hz)	δ_H_ (*J* in Hz)	δ_H_ (*J* in Hz)	δ_H_ (*J* in Hz)
1α	2.97ddt (19.2, 5.0, 1.8)	2.96 br dd (19.2, 4.8)	2.91 br dd (19.2, 4.8)	3.04 ddt (19.2, 4.8, 1.8)	2.96 ddt (19.2, 4.8, 2.1)	3.01 br dd (19.0, 5.4)	4.69 ddd (7.8, 2.6, 1.1)
1β	2.44 ddt (19.2, 7.8, 2.0)	2.42 br dd (19.2, 7.2)	2.39 ddt (19.2, 7.2, 3.0)	2.52 ddt (19.2, 7.2, 2.4)	2.45 ddt (19.2, 7.2, 1.8)	2.32 br dd (19.0, 8.4)	
2	3.69 dt (5.0, 7.8)	3.66 dt (4.8, 7.2)	3.64 dt (4.8, 7.2)	3.76 dt (4.8, 7.2)	3.71 dt (5.4, 7.2)	3.5 dt (5.4, 8.4)	3.5 dd (11.4, 7.8)
3	1.85 m	1.81 m	1.80 m	1.92 m	1.86 m	1.85 m	1.85 m
4α	2.89 ddt (19.5, 6.6, 1.8)	2.82 dd (19.8, 6.0)	2.81 dd (19.8, 6.0)	2.92 ddt (19.8, 6.0, 1.8)	2.89 ddt (19.8, 5.4, 1.8)	3.11 dd (18.6, 5.4)	2.94 ddd (19.8, 4.8, 0.6)
4β	2.24 ddq (19.5, 7.8, 1.8)	2.18 br dd (19.8, 8.4)	2.18 ddq (19.8, 7.8, 1.8)	2.28 ddt (19.8, 7.8, 2.4)	2.26 ddt (19.8, 8.4, 1.8)	2.55 dd (18.6, 7.8)	2.16 ddd (19.8, 11.4, 3.6)
5		7.39 s					
8	7.42 s					12.9 s	7.24 s
CH_3_ (3)	1.07 br d (7.8)	1.03 d (6.6)	1.01 d (6.6)	1.12 d (6.6)	1.07 d (7.2)	1.07 d (6.6)	1.22 d (6.6)
OH (5)				13.2 s		12.9 s	12.1 s
OCH_3_ (5)	3.90 s		3.94 s ^c^		3.85 s		
OCH_3_ (6)	3.97 s	3.88 s	3.84 s ^c^	4.07 s	4.01 s	4.05 s	3.99 s
OCH_3_ (7)	3.93 s	3.93 s	3.84 s ^c^	4.06 s	4.02 s	4.04 s	4.00 s
OCH_3_ (8)		3.86 s	3.94 s ^c^	3.90 s			
OH(8)					13.1 s	12. 9 s	

^a^ The chemical shifts in δ values (ppm) from tetramethylsilane (TMS); ^b^ Readout [δ_H_ (*J* in Hz)] in CDCl_3_ at 600 MHz; ^c^ overlapped.

### 2.1. Chemical Structure of Prisconnatanones C to I (Compounds ***1**–**7***)

Compound **1** formed yellow crystals, and its molecular formula was C_18_H_20_O_6_, as determined by the high-resolution electrospray ionization mass spectrometry (HRESIMS) [M + Na]^+^ ion at *m*/*z* 355.1152 and ^13^C-NMR spectroscopy (Table 1), showing nine degrees of unsaturation. The infrared (IR) spectrum displayed the presence of hydroxy (3441 cm^−1^), conjugated carbonyl (1655 cm^−1^), and aromatic ring groups (1610, 1577, and 1459 cm^−1^) in this compound. Based on a previous study [11] the UV spectrum suggested that there might be an anthraquinone chromophore, according to the presence of absorption peaks at 210, 268, 362 nm. The ^13^C-NMR spectrum revealed 18 characteristic signals indicating two quinone carbonyl groups (δ_C_ 184.2, 183.3) on positions 9 and 10, eight aromatic or olefinie carbons (δ_C_ 157.3, 154.3, 147.9, 145.0, 139.8, 129.7, 119.7, 106.1), five sp^3^ carbons (δ_C_ 71.0, 34.2, 30.9, 29.9, 17.5) and three methoxy groups (δ_C_ 61.8, 61.6, 56.6) (Table 1). The ^1^H-NMR data showed an aromatic proton δ_H_ 7.42 (1H, s), and three methoxyls (δ_H_ 3.97, 3.93, 3.90, every 3H, s) (Table 2). In the comparison with the published data of prisconnatanone A [9], both the ^1^H-NMR and ^13^C-NMR data were similar, suggesting that compound **1** was also a tetrahydroanthraquinone. Analysis of 1D NMR, ^1^H-^1^H COSY, and HSQC data revealed the presence of one 2-hydroxy-3-methylbutane unit and one pentasubstituted nephthoquinone moiety. The methyl (δ_C_ 17.4) was confirmed to be at C-3 by ^1^H-^1^H COSY (Figure 2), cross-peaks (from H-3 to H_3_-Me, H-2, H-4, from H_2_-1 to H-2) and HMBC correlations (from H_3_-Me to C-2, C-3, and C-4) (Figure 2). The aromatic proton δ_H_ 7.42 (1H, s) showed HMBC correlations with C-9 (δ 184.2), C-12 (δ 119.7), C-7 (δ 157.3), suggesting that C-8 was unsubstituted and the three methoxy groups (δ_C_ 61.8, 61.6, 56.6) were located at C-5, C-6 and C-7. Therefore, the planar structure of compound **1** was identified as 1,2,3,4-tetrahydro-2-hydroxy-5,6,7-trimethoxy-3-methylanthracene-9,10-dione (Figure 1).

**Figure 4:**
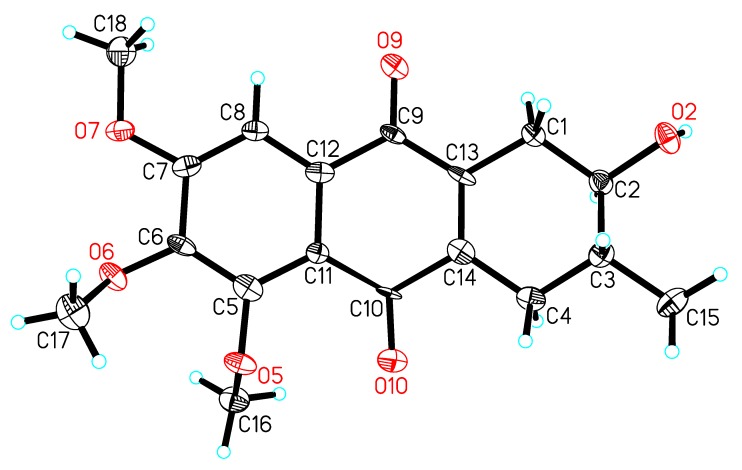
X-ray crystal structure of compound **1**.

**Figure 5:**
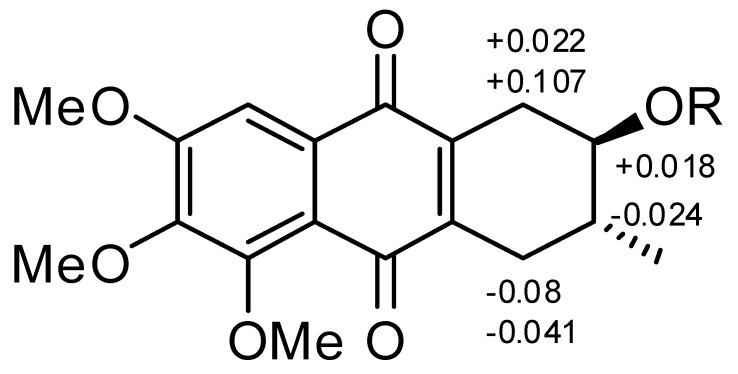
Δδ (δ_S_-δ_R_) values (in ppm) for the MTPA esters of **1**. 1**a** R = (S)-MTPA; 1**b** R = (R) − MTPA.

A single crystal of compound **1** was obtained by slow crystallization in methanol and water, and was mounted on a X-ray diffractometer equipped with Cu Kα monochromated radiation, which allowed the compound structure to be unequivocally determined with OH-2, Me-3 in a *trans* relationship (Figure 4). The absolute configuration of the chiral centers at C-2 and C-3 of **1** was assigned as 2*R* and 3*R* by using Hooft methods, which were further confirmed by application of the advanced Mosher’s method (Figure 5). Therefore, compound **1** was trivially named as prisconnatanone C and unequivocally identified as (2*R*,3*R*)-1,2,3,4-tetrahydro-2α-hydroxy-5,6,7-trimethoxy-3β-methylanthracene-9,10-dione (Figure 1). To the best of our knowledge, this is the first time the absolute configuration of a tetrahydroanthraquinone from *P. connata* has been reported.

Compound **2** showed a pseudomolecular [M + Na]^+^ ion peak at *m/z* 355.1156, indicating the same molecular formula C_18_H_20_O_6_ as **1** based on the HRESIMS data. By comparison of their 1D NMR (Table 1 and Table 2), there were slight differences in two aromatic carbons and two methylene groups (Figure 1): **1** with (δ_C_ 145.0, 139.8, 30.9, 29.9) and **2** with (δ_C_ 143.6, 141.2, 31.3, 29.4). Further, HMBC correlations from H-5 to C-6, C-10 and C-11 were observed in **2**, revealing that the C-5 was unsubstituted and the three methoxy groups (δ_C_ 56.6, 61.6, 61.8) were located at C-6, C-7 and C-8 (Figure 2). Besides, a ^3^*J*_H-2,H-3_ = 7.2 Hz coupling constant, in conjunction with a ROESY correlation were observed between H-1 and H_ax_-3 and between H-2 and H_ax_-4, indicating the axial–axial relationship for these two protons and both OH-2, Me-3 in equatorial positions (Figure 3). The optical rotation (ORD) of **2**, ${\left[\alpha \right]}_{D}^{21}$ = −55.7 (*c* = 0.16, MeOH), had the similar sign and magnitude as seen in **1**, ${\left[\alpha \right]}_{D}^{21}$ = −61.7 (*c* = 0.2, MeOH), suggesting the same absolute configurations at C-2 and C-3. Thus, compound **2** was identified as (2*R*,3*R*)-1,2,3,4-tetrahydro-2α-hydroxy-6,7,8-trimethoxy-3β-methylanthracene-9,10-dione (Figure 1) and named prisconnatanone D. 

Compound **3** was a yellow power with a pseudomolecular [M + Na]^+^ ion at *m/z* 385.1258, indicating a molecular formula of C_19_H_22_O_7_. The ^1^H- and ^13^C-NMR spectra (Table 1 and Table 2) were similar to those of **2**, except for the presence of an additional methoxy group (Figure 1). There was no aromatic proton in the ^1^H-NMR spectrum, suggesting that the four positions at aromatic ring of compound **3** were substituted and the extra methoxy was connected to C-8 (Figure 1). The absolute configurations at C-2 and C-3 were determined as *R* by the similar ORD between **2**, ${\left[\alpha \right]}_{D}^{21}$ = −55.7 (*c* = 0.16, MeOH), and **3**, ${\left[\alpha \right]}_{D}^{21}$ = −57.8 (*c* = 0.2, MeOH). By combined analysis of the 2D NMR spectra (^1^H-^1^H COSY, HSQC, HMBC and ROESY) (Figure 2 and Figure 3), the structure of **3** was identified as 1,2,3,4-tetrahydro-2α-hydroxy-5,6,7,8-tetramethoxy-3β-methylanthracene-9,10-dione (Figure 1) and it was named prisconnatanone E.

Compound **4** was a red, amorphous powder with a negative ion [M − H]^−^ peak at *m*/*z* 347.1137, corresponding to the molecular formula C_18_H_20_O_7_ on the basis of its ^13^C-NMR spectroscopic and HRESIMS data, and indicating the absence of a methylene as compared with **3**. The ^1^H-NMR (Table 2) spectra of **4** were similar to **3** except for a characteristic chelated hydroxyl group observed as a sharp singlet at δ_H_ 13.2 instead of a methoxy. Furthermore, the observation of HMBC correlations (from OH-5 to C-5, C-6, C-10 and C-11) suggested that the phenolic hydroxyl was connected to C-5 (Figure 1). According to the similar ORD between compound **2**, ${\left[\alpha \right]}_{D}^{21}$ = −55.7 (*c* = 0.16, MeOH), and compound **4**, ${\left[\alpha \right]}_{D}^{21}$ = −68.4 (*c* = 0.105, MeOH), the absolute configurations at C-2 and C-3 were deduced as *R*. Thus, this compound was named as prisconnatanone F, and based on combination of ^1^H-^1^H COSY, HSQC, HMBC, and ROESY (Figure 2 and Figure 3), it was identified as 1,2,3,4-tetrahydro-2α,5-dihydroxy-6,7,8-trimethoxy-3β-methylanthracene-9,10-dione (Figure 1).

Compound **5** was a red powder with a pseudomolecular ion at *m*/*z* 371.1114 ([M + Na]^+^), suggesting a molecular formula of C_18_H_20_O_7_ based on the HRESIMS data, which was the same as that of **4**. From carefully comparison of the differences between **5** and **4** as well as between **1** and **2**, there was just a small distinction between the two aromatic carbons and two methylenes (Figure 1). Based on the data of **2**, the phenolic hydroxy was placed at C-8, which was supported by HMBC correlations (from OH-8 to C-7, C-8, C-9 and C-12), and also there was similar ORD between **2**, ${\left[\alpha \right]}_{D}^{21}$ = −55.7 (*c* = 0.16, MeOH), and **5**, ${\left[\alpha \right]}_{D}^{21}$ = −61.3 (*c* = 0.20, MeOH), suggesting the absolute configurations at C-2 and C-3 were also *R* (Figure 2). The relative configuration of compound **5** was determined to be the same as that of **4** based on ROESY experiments and coupling constants, suggesting that **5** was isomeric with **4**. Thus, compound **5** was named prisconnatanone G and identified as 1,2,3,4-tetrahydro-2α,8-dihydroxy-5,6,7-trimethoxy-3β-methylanthracene-9,10-dione (Figure 1).

Compound **6** was red, amorphous powder with a pseudomolecular ion [M + Na]^+^ at *m*/*z* 357.0941, indicating a molecular formula of C_17_H_18_O_7_ based on the HRESIMS. Comparison of its NMR spectra with those of the tetrahydroanthraquinones indicated that the ^1^H- and ^13^C-NMR data of **6** (Table 1 and Table 2) were similar to those of **4**, except that a hydroxyl at δ_H_ 12.89 in **6** replaced a methoxyl in **4**. 2D NMR analysis indicated that the hydroxyl group was located at C-5. The absolute configurations 2*R* and 3*R* were determined according to the similar ORD between **1**, ${\left[\alpha \right]}_{D}^{21}$ = −61.7 (*c* = 0.20, MeOH), and **6**, ${\left[\alpha \right]}_{D}^{21}$ = −45.3 (*c* = 0.075, MeOH). Therefore, the structure of **6** was named as prisconnatanone H and identified as 1,2,3,4-tetrahydro-2α,5,8-trihydroxy-6,7-dimethoxy-3β-methylanthracene-9, 10-dione (Figure 1).

**Figure 6:**
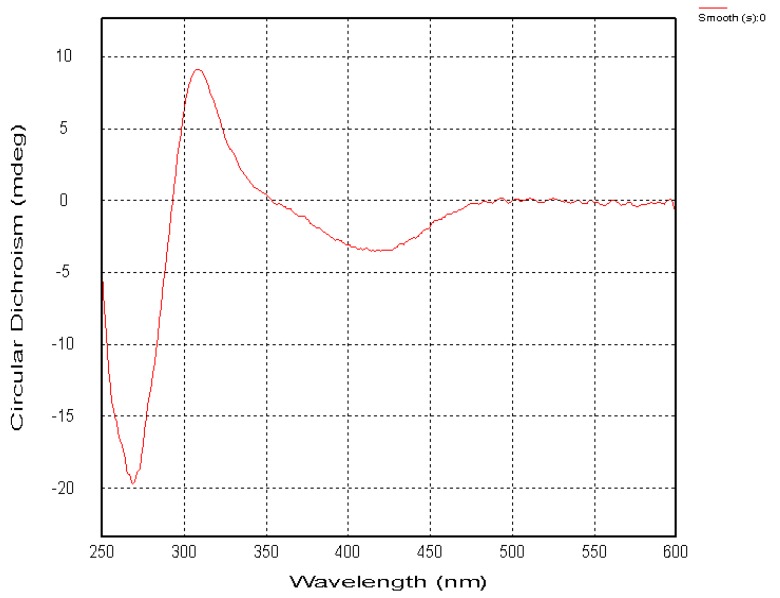
The circular dichroism (CD) spectrum of compound **7** in DMSO.

**Figure 7:**
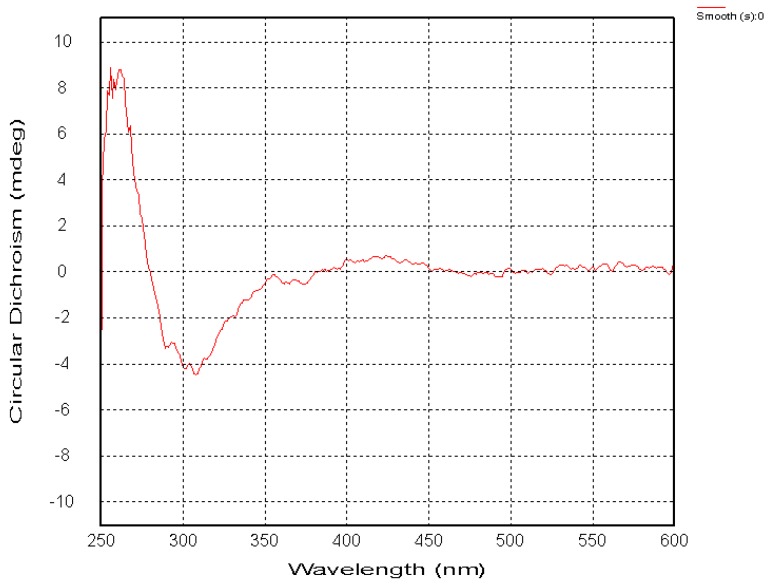
The circular dichroism (CD) spectrum of compound **7** with Mo_2_OAc_4_ in DMSO.

Compound **7** was a reddish brown, amorphous solid with a pseudomolecular ion [M + Na]^+^ peak at *m/z* 357.0948, suggesting the molecular formula C_17_H_18_O_7_ based on HRESIMS data. The ^1^H- and ^13^C-NMR (Table 1 and Table 2) spectra were similar to those of **6**, except of the absence of a sharp singlet hydroxyl signal (Figure 1). Besides, two *O*-bearing methines δ 76.7 (C-1), and 73.0 (C-2), and only one methylene group δ 31.1 (C-4) were detected at a high field in the ^13^C-NMR, indicating the absence of the hydroxyl moved to C-1 (Figure 1). The HMBC correlations (from OH-5 to C-5, C-6, C-10, and C-11, from H-8 to C-7, C-9 and C-12) implied that phenolic hydroxyl δ_C_ 12.1 was located at C-5, and two methoxy groups (δ_C_ 61.3, 56.7) were attached to C-6 and C-7 (Figure 2). Meanwhile, the three substituents (OH-1, OH-2, Me-3) were identified at equatorial locations by the two coupling constants ^3^*J*_H-1,H-2_ = 7.8 Hz and ^3^*J*_H-2,H-3_ = 11.2 Hz. Based on these data, compound **7** was named prisconnatanone I and identified as 1,2,3,4-tetrahydro-1α,2β,5-trihydroxy-6,7-dimethoxy-3β-methylanthracene-9,10-dione (Figure 1). The absolute configurations of this compound were further determined by using circular dichroism (CD) with the Mo_2_OAc_4_ method (Figure 6 and Figure 7). A negative Cotton effect (CE) at 312 nm in the CD spectrum of **7** (Figure 7) with Mo_2_OAc_4_ was observed, suggesting that the absolute configuration of C-1, C-2 and C-3 could be assigned as *R*,*R*,*S*, respectively.

### 2.2. Structure-Dependent Cytotoxicity of Prisconnatanones C to I (***1**–**7***) against Tumor Cell Growth

**Figure 8:**
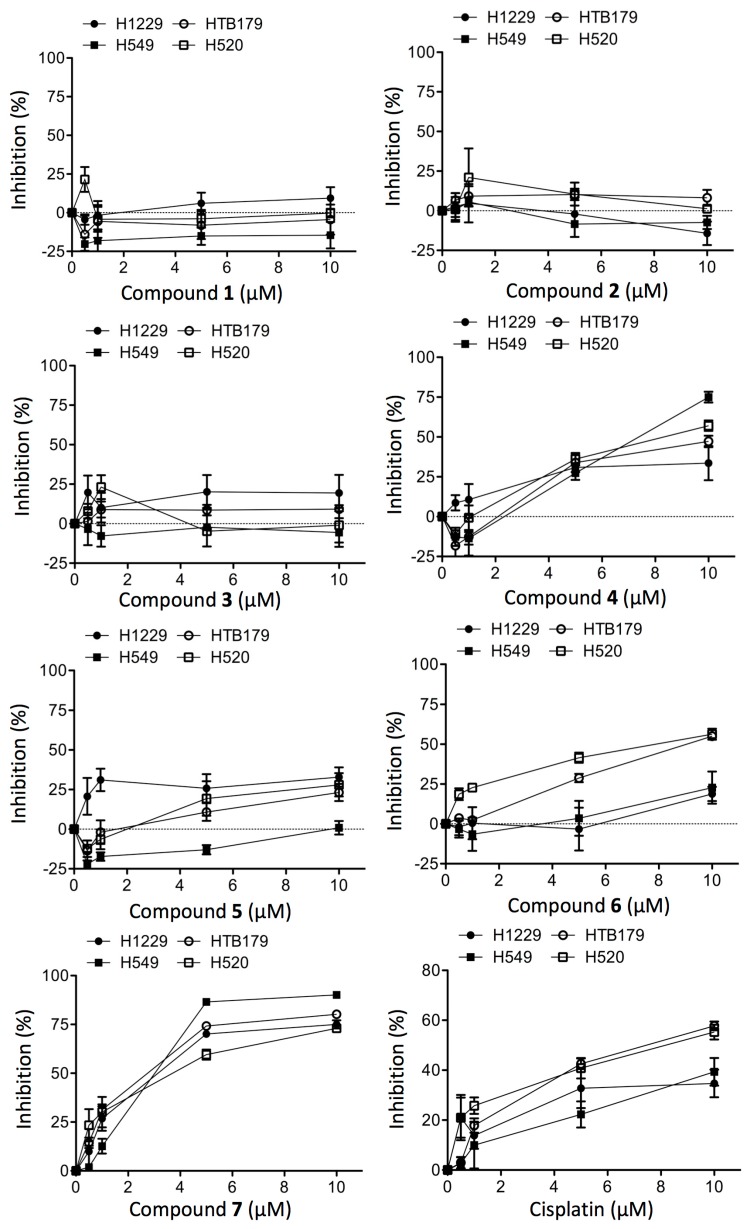
Inhibition of cell growth by compounds **1**–**7** as compared to cisplatin control in cultured human lung tumor.

The current study led to the identification of seven new compounds **1**−**7**, which belonged to a class of tetrahydroanthraquinones by their chemical structure. Finally, the cytotoxicity of all of these compounds **1**–**7** at a range of up to 10 µM was tested against a panel of four non-small cell lung tumor cell lines (H1229, HTB179, A549 and H520). As shown in Figure 8, compounds **1**–**3** did not show any inhibitory activity at all to any of the cell lines, while the inhibitory activities of compounds **4**–**6** were relatively low, and compound **7** showed the most potent cytotoxicity among these compounds against the growth of these lung tumor cells. Based on the IC_50_ values of these compounds against the growth of lung tumor cells (Table 3), their cytotoxicity could be ranked in the order of decreasing inhibitory activity as follows: Compound **7** > **6** = **4** > **5** > **1** = **2** = **3**. The activity profiles of these compounds implied that the positions of hydroxyl groups (C-5 and C-8) might be necessary for their antitumor potency, and hydroxylation at C-1 could significantly enhance its cytotoxic activity; however, the underlying mechanisms remain further investigation. Cisplatin is one of first-line drugs in the treatment of lung cancer [12]. Comparing the cytotoxicity of this drug (IC_50_: >20 µM in H1229; 7.5 µM in HTB179; 14.5 µM in H549; and 8 µM in H520) with those of the isolated compounds (Figure 8, Table 3), the cytotoxicity of **7** was higher than that of cisplatin against this panel of lung tumor cells.

**Table 3:** Cytotoxic activity (mean value of IC_50_ at µM) of compounds **1**−**7**.

Compound	H1229	HTB179	H549	H520
**1**	Nil	Nil	Nil	Nil
**2**	Nil	Nil	Nil	Nil
**3**	Nil	Nil	Nil	Nil
**4**	>20	9.8	>20	9.5
**5**	>20	>20	>20	>20
**6**	>20	13	8	9.5
**7**	3.0	2.7	3.0	3.9

Data are presented as a mean value (IC_50_ at µM) of 2–3 separate experiments. Nil: no effect was found.

The tumor cell cultures were incubated in the absence (untreated control) or presence of 0.5, 1, 5 and 10 µM of a purified compound or cisplatin for 48 h. The cell viabilities at the end of incubation were determined with MTT assay. Data are presented as means ± standard derivation (SD) of six determinants in a typical experiment that was repeated 2–3 times. Dot line: *x*-axis (*y* = 0).

## 3. Experimental Section

### 3.1. General Experimental Procedures

The chemical analyses of purified compounds were performed as follows: optical rotations were examined using a Jasco P-1020 polarimeter (Jasco Analytical Instruments, Easton, MD, USA) in either MeOH or CHCl_3_, the UV spectra using a Shimadzu UV-2401PC (Shimadzu China Co., Beijing, China) with MeOH, the CD spectra using a Chirascan spectropolarimeter (Applied Photophysics, Surrey, UK), the IR spectra using a Bruker Tensor 27 Fourier transform infrared spectrometer with KBr pellets (Bruker Beijing Scientific Tech Co., Beijing, China), the ^1^H (600 MHz), ^13^C (150 MHz) and 2D NMR using Bruker AM-400 and DR-600 instruments (Bruker Beijing Scientific Tech Co.) with TMS as an internal standard in CDCl_3_, the ESIMS using a Bruker HCT/E spectrometer (Bruker Beijing Scientific Tech Co.) and the HREIMS using Waters Autospec Premier P776 spectrometer (Waters Co., Milford, MA, USA). Column chromatography was performed on both silica gel (60–80; 200–300; 300–400 mesh) (Qingdao Marine Chemical Group Co., Qingdao, China) and Sephadex LH-20 (GE Healthcare LifeSciences, Little Chalfont, UK), in which the fractions were monitored by TLC (GF254, Qingdao Marine Chemical Co.). X-ray diffraction data were collected using an Aglient Technologies Gemini A Ultra system (Agilent Tech, Santa Clara, CA, USA).

### 3.2. Plant Material and Drug

HG was collected in June, 2011 from Nanning, Guangxi, China, and identified by Dr. Tao Chen, one of the co-authors. An authenticated voucher specimen (No. CT20110601) was deposited in the herbarium collection (SZG) at the Fairy Lake Botanical Garden (Chinese Academy of Sciences, Shenzhen, China). Cisplatin was purchased from the Pharmacy Services at Vancouver General Hospital (Vancouver, BC, Canada).

### 3.3. Extraction, Isolation and Identification

HG extract was prepared from the dried and powdered HG (15 kg) with 95% EtOH (3 × 40 L). The solvent was evaporated under reduced pressure until a paste-like extract was formed. The remaining residue (280 g) was suspended in H_2_O, and respectively partitioned using petroleum ether, EtOAc, and BuOH. Next, the EtOAc part (94 g) was submitted to silica gel column chromatography (200–300 mesh) with a gradient elution of PE–EtOAc (from 5:1 to 1:1), and fourteen fractions (Fr.) were collected. All compounds **1**–**7** were purified from Fr. 12 to Fr. 14.

Fr. 12 (containing **1** and **2**) was fractionated using a silica gel column (200–300 mesh) with chloroform–methanol (1000:8) to give fractions 11A–C, followed by further separation of 11A using Sephadex LH-20 column (MeOH), pressurized silica gel column (300–400 mesh) with methanol–acetone–petroleum ether (1:10:95), and then preparative HPLC (MeCN–H_2_O, 60:40) to obtain **1** (73.2 mg, t_R_ 28.32 min) and **2** (17.7 mg, t_R_ 32.12 min).

Fr. 13 (containing **4** and **5**) was subjected to silica gel column chromatography (200–300 mesh) with chloroform-methanol (1000:10) to give fractions 13A–F. Fraction 13B was further separated using a Sephadex LH-20 column (MeOH) and then a pressurized silica gel column (300–400 mesh) with methanol–acetone–petroleum ether (1:10:85) to obtain **4** (34.4 mg). Fraction 13D was further purified by using a Sephadex LH-20 column (MeOH), and a pressurized silica gel column (300–400 mesh) with methanol–acetone–petroleum ether (1:10:80), followed by preparative HPLC (MeCN–H_2_O, 30:70) to obtain **5** (9.1 mg, t_R_ 14.12 min).

Fr. 14 (containing **6**, **7** and **3**) was fractionated by using silica gel column chromatography (200–300 mesh) with chloroform–methanol (1000:13) as eluents to afford fractions 14A-F, of which fraction 14B was further purified by using Sephadex LH-20 column chromatography (MeOH), pressurized silica gel column chromatography (300–400 mesh) with methanol–acetone–petroleum ether (1:10:75), and then by preparative HPLC (MeOH–H_2_O, 70:30) to obtain **6** (10.3 mg, t_R_ 15.32 min). Fraction 14F was purified by using Sephadex LH-20 column chromatography (MeOH), and pressurized silica gel column chromatography (300–400 mesh) with methanol–acetone–petroleum ether (1:10:70) to obtain **7** (45.9 mg). Similarly, **3** (7.7 mg) was obtained from fraction 14C by using Sephadex LH-20 column chromatography with MeOH as eluent.

### 3.4. Structural Analyses

*1,2,3,4-Tetrahydro-2-hydroxy-5,6,7-trimethoxy-3-methylanthracene-9,10-dione (Prisconnatanone C, ***1***, C_18_H_20_O_6_)*: yellow acicular crystals; ${\left[\alpha \right]}_{\text{D}}^{21}$ = −61.7 (*c* = 0.2, MeOH); UV (MeOH) λ_max_ (log ε) 362 (2.81), 268 (3.68), 210 (3.80) nm; IR (KBr) ν_max_ 3441, 1655, 1577, 1459 cm^−1^; ^1^H- and ^13^C-NMR, see Table 1 and Table 2; ESIMS (positive) *m*/*z* 355 [M + Na]^+^; HREIMS (positive) *m*/*z* 355.1152 [M + Na]^+^ (calculated for C_18_H_20_O_6_, 355.1158).

*1,2,3,4-Tetrahydro-2α-hydroxy-6,7,8-trimethoxy-3β-methylanthracene-9,10-dione (Prisconnatanone D,* **2***, C_18_H_20_O_6_)*: yellow powder; ${\left[\alpha \right]}_{\text{D}}^{21}$ = −55.7 (*c* = 0.16, MeOH); UV (MeOH) λ_max_ (log ε) 361 (2.56), 267.5 (3.43), 209.5 (3.55) nm; IR (KBr) ν_max_ 3452, 1653, 1577, 1484 cm^−1^; ^1^H- and ^13^C-NMR, see Table 1 and Table 2; ESIMS (positive) *m*/*z* 355 [M + Na]^+^; HREIMS (positive) *m*/*z* 355.1156 [M + Na]^+^ (calculated for C_18_H_20_O_6_, 355.1158).

*1,2,3,4-Tetrahydro-2α-hydroxy-5,6,7,8-tetramethoxy-3β-methylanthracene-9,10-dione (Prisconnatanone E,* **3***, C_19_H_22_O_7_)*: yellow powder; ${\left[\alpha \right]}_{\text{D}}^{21}$ = −57.8 (*c* = 0.2, MeOH); UV (MeOH) λ_max_ (log ε) 375 (2.59), 262 (3.25), 215 (3.45) nm; IR (KBr) ν_max_ 3433, 1654, 1546, 1465 cm^−1^; ^1^H- and ^13^C-NMR, see Table 1 and Table 2; ESIMS (positive) *m*/*z* 355 [M + Na]^+^; HREIMS (positive) *m*/*z* 385.1258 [M + Na]^+^ (calculated for C_19_H_22_O_7_, 385.1263).

*1,2,3,4-Tetrahydro-2α,5-dihydroxy-6,7,8-trimethoxy-3β-methylanthracene-9,10-dione (Prisconnatanone F,* **4***, C_18_H_20_O_7_)*: red powder; ${\left[\alpha \right]}_{\text{D}}^{21}$ = −68.4 (*c* = 0.105, MeOH); UV (MeOH) λ_max_ (log ε) 433 (2.95), 289 (3.17), 262.5 (3.32), 224 (3.73), 194.5 (3.46) nm; IR (KBr) ν_max_ 3449, 1640, 1611, 1454 cm^−1^; ^1^H- and ^13^C- NMR, see Table 1 and Table 2; ESIMS (positive) *m*/*z* 371 [M + Na]^+^; HREIMS (negative) *m*/*z* 347.1137 [M − H]^+^ (calculated for C_18_H_20_O_7_, 347.1131).

*1,2,3,4-Tetrahydro-2α,8-dihydroxy-5,6,7-trimethoxy-3β-methylanthracene-9,10-dione (Prisconnatanone G,* **5***, C_18_H_20_O_7_)*: red powder; ${\left[\alpha \right]}_{\text{D}}^{21}$ = −61.3 (*c* = 0.20, MeOH); UV (MeOH) λ_max_ (log ε) 430.5 (2.72), 289.5 (2.94), 261.5 (3.08), 224 (3.51) nm; IR (KBr) ν_max_ 3432, 1641, 1611, 1454 cm^−1^; ^1^H- and ^13^C-NMR, see Table 1 and Table 2; ESIMS (positive) *m*/*z* 371 [M + Na]^+^; HREIMS (positive) *m*/*z* 371.1114 [M + Na]^+^ (calculated for C_18_H_20_O_7_, 371.1107).

*1,2,3,4-Tetrahydro-2α,5,8-trihydroxy-6,7-dimethoxy-3β-methylanthracene-9,10-dione (Prisconnatanone H,* **6***, C_17_H_18_O_7_)*: red powder; ${\left[\alpha \right]}_{\text{D}}^{21}$ = −45.3 (*c* = 0.075, MeOH); UV (MeOH) λ_max_ (log ε) 572 (2.45), 532.5 (2.83), 498.5 (2.88), 313.5(2.84), 233(3.36), 209.5(3.32) nm; IR (KBr) ν_max_ 3443, 1630, 1598, 1454 cm^−1^; ^1^H- and ^13^C-NMR, see Table 1 and Table 2; ESIMS (positive) *m*/*z* 357 [M + Na]^+^; HREIMS (positive) *m*/*z* 357.0941 [M + Na]^+^ (calculated for C_17_H_18_O_7_, 357.0950).

*1,2,3,4-Tetrahydro-1α,2β,5-trihydroxy-6,7-dimethoxy-3β-methylanthracene-9,10-dione (Prisconnatanone I,* **7***, C_17_H_18_O_7_)*: reddish brown solid; ${\left[\alpha \right]}_{\text{D}}^{21}$ = −360 (*c* = 0.11, CHCl_3_); UV (MeOH) λ_max_ (log ε) 416 (2.80), 267.5 (3.48), 218.5 (3.66) nm; IR (KBr) ν_max_ 3439, 1631, 1579, 1451 cm^−1^; ^1^H- and ^13^C-NMR, see Table 1 and Table 2; ESIMS (positive) *m*/*z* 357 [M + Na]^+^; HREIMS (positive) *m*/*z* 357.0948 [M + Na]^+^ (calculated for C_17_H_18_O_7_, 357.0950).

### 3.5. Crystal Data for ***1***

4(C_18_H_20_O_6_)·CH_4_O·2(H_2_O), *M* = 1397.43, orthorhombic, *a* = 14.2508(3) Å, *b* = 44.4928(11) Å, *c* = 10.7064(3) Å, α = 90.00°, β = 90.00°, γ = 90.0°, *V* = 6788.5(3) Å^3^, *T* = 100(2) K, space group *P*21212, *Z* = 4, µ(CuKα) = 0.874 mm^−1^, 30337 reflections measured, 11554 independent reflections (*R_int_* = 0.1114). Here were the final values: *R_1_* = 0.1143 (*I* > 2σ(*I*)), *wR*(*F*^2^) = 0.2875 (*I* > 2σ(*I*)), and from all data: *R_1_* = 0.1913, and *wR*(*F*^2^) = 0.3513. The goodness of fit on *F*^2^ was 1.056. Flack parameter = 0.2(4). The Hooft parameter was 0.03(16) for 4723 Bijvoet pairs. The supplementary crystallographic data for this paper was deposited in the Cambridge Crystallographic Data Centre (No: 1419549). These data can be obtained free of charge via http://www.ccdc.cam.ac.uk/conts/retrieving.html (or from the CCDC, 12 Union Road, Cambridge CB2 1EZ, UK; Fax: +44 1223 336033; E-mail: deposit@ccdc.cam.ac.uk).

### 3.6. Esterification of Prisconnatanone C (***1***) with (R)- and (S)-MTPA

Two portions of prisconnatanone C (**1**), 12 mg, 0.036 mmol each) were treated with (*R*)- and (*S*)-MTPA in C_2_H_2_ with dimethylaminopyridine and *N*,*N*′-dicyclohexylcarbodiimide at room temperature and pressure, respectively. These two reactions were conducted in parallel, and their progress was monitored by using TLC. After the reaction was completed, and then the solvent was evaporated, the resultant materials were submitted to silica gel column chromatography (200–300 mesh) with PE–EtOAc (2:1), and further purified by using preparative HPLC (MeCN–H_2_O, 80:30), from which the (*S*)-MTPA (1**a**, t_R_ 13.21 min) and (*R*)-MTPA (**1b**, t_R_ 12.54 min) esters of prisconnatanone C (**1**) were collected.

### 3.7. Tumor Cell Cultures

All the lung tumor cell lines were obtained from the Vancouver Prostate Centre (Vancouver, BC, Canada) and were cultured at 37 °C under 5% of CO_2_ atmosphere in following culture media (Thermo Scientific, Rockford, IL, USA): H1299, H520 and A549 were cultured in Dulbecco’s modified Eagle’s Medium (DMEM), and HTB-179 was grown in Roswell Park Memorial Institute (RPMI) 1640 medium. Both DMEM and RPMI 1640 media were supplemented with heat-inactivated fetal bovine serum (FBS, 10%) and antibiotic mixture (100 U/mL penicillin and 100 µg/mL streptomycin) (Sigma-Aldrich Canada, Oakville, ON, Canada).

### 3.8. Cell Viability Assay

The effects of all of the purified compounds **1**–**7** on lung tumor cell growth or viability, expressed as the percentage of inhibition, were determined using a MTT (3-[4,5-dimethylthiazaol- 2-yl]-2,5-diphenyltetrazolium bromide, Sigma-Aldrich Canada) assay as described previously [13]. In brief, tumor cells in 96-well plates at a density of 2.5–4.0 × 10^3^ cells/well depending cell types were incubated in the absence (untreated control) or the presence of various concentrations of the compounds (0.5 to 10 µM) for 48 h, MTT solution (5.0 mg/mL in PBS) was added (10 µL/well), and plates were incubated for another 4 h at 37 °C. The purple formazan crystals were dissolved in 100 µL/well of dimethyl sulfoxide (DMSO, Sigma-Aldrich Canada) for 5 min, and the absorbance (OD) was quantified at a 560 nm wavelength using an ELx808 Ultra Microplate Reader (BioTek, Winooski, VT, USA). Each experiment was repeated 2–3 times. Cell growth inhibition in drug-treated cultures against nondrug-treated control was calculated as follows: Inhibition (%) = (Control − Drug-treated)/Control × 100%. The half maximal inhibitory concentration (IC_50_) was calculated based on the cytotoxicity curves as described previously [14].

## 4. Conclusions

In conclusion, this study described the structural analyses of prisconnatanones C to I (compounds **1**–**7**) isolated from the root of *P. connata* and confirmed that these new natural compounds belonged to the rare tetrahydroanthraquinone structural class. In the cytotoxicity assay with lung tumor cells, the relationship of the chemical structure of these compounds with their activities suggested that the chelated phenolic hydroxyls might be the key functional group for their inhibitory activity as compounds **4**–**7** with chelated phenolic hydroxyls showed activity, while those without the group (compounds **1**–**3**) didn’t have any activity. Furthermore, because the most potent activity was found in compound **7**, it was suggested that the activity might be enhanced by the numbers of oxygen substituent at the benzene ring of the compounds.

## References

[1] Compendium (1992). Compendium of Chinese Traditional Herbal Drugs.

[2] Zhou C.Y., Deng J.G. (2006). Progress in chemical and pharmacological studies on *Prismatomeris tetrandra*. J. Guangxi Tradit. Chin. Med. Univ..

[3] Ruan Y.Z. (1988). Notes on the Genus *Prismatomeris* Thw. (Rubiaceae) of China. J. Syst. Evol. (Acta Phytotax Sin.).

[4] Tu D.J., Pang Z.H., Bi N.J. (1981). Studies on constituents of *Prismatomeris tetrandra* (Roxb) K Schum. Yao Xue Xue Bao.

[5] Jiang J.S., Feng Z.M., Zhang P.C. (2005). Chemical constituents from root of *Prismatomeris tetrandra*. Zhongguo Zhong Yao Za Zhi.

[6] Feng Z.M., Jiang J.S., Wang Y.H., Zhang P.C. (2005). Anthraquinones from the roots of *Prismatomeris tetrandra*. Chem. Pharm. Bull..

[7] Zhang C.L., Guan H., Xi P.Z., Deng T., Gao J.M. (2010). Anthraquinones from the roots of *Prismatomeris tetrandra*. Nat. Prod. Commun..

[8] Hao J., Feng S.X., Qiu S.X., Chen T. (2011). Anthraquinone glycosides from the roots of *Prismatomeris connata*. Chin. J. Nat. Med..

[9] Feng S.X., Hao J., Chen T., Qiu S.X. (2011). A new anthraquinone and two new tetrahydroanthraquinone from the roots of *Prismatomeris connata*. Helv. Chim. Acta.

[10] Feng S., Bai J., Qiu S., Li Y., Chen T. (2012). Iridoid and phenolic glycosides from the roots of *Prismatomeris connata*. Nat. Prod. Commun..

[11] Suemitsu R., Yamada Y., Sano T., Yamashita K. (1984). Phytotoxic activities of altersolanol-a, Altersolanol-b and dactylariol, and activities of altersolanol-a against some microorganisms. Agric. Biol. Chem. Tokyo.

[12] Ardizzoni A., Boni L., Tiseo M., Fossella F.V., Schiller J.H., Paesmans M., Radosavlievic D., Paccaqnella A., Zatloukal P., Mazzanti P. (2007). Cisplatin-versus carboplatin-based chemotherapy in first-line treatment of advanced non-small-cell lung cancer: An individual patient data meta-analysis. J. Natl. Cancer Inst..

[13] Feng S.X., Guan Q., Chen T., Du C. (2012). *In vitro* activities of 3-hydroxy-1,5,6-trimethoxy-2-methyl-9,10-anthraquinone against non-small cell lung carcinoma. Arch. Pharm. Res..

[14] Bliss C. (1935). The calculation of the dose-mortality curve. Ann. Appl. Biol..

